# Supplementary material for: The serine proteases dipeptidyl-peptidase 4 and urokinase are key molecules in human and mouse scar formation
Source: Nat Commun. 2021 Oct 29;12:6242. doi: 10.1038/s41467-021-26495-2 (PMC8556235; doi:10.1038/s41467-021-26495-2)
Supplement: Supplementary file 1 — Supplementary Information [file 41467_2021_26495_MOESM1_ESM.pdf]

Supplementary Information

The serine proteases dipeptidyl-peptidase 4 and urokinase are key molecules in human and mouse scar formation

Vera Vorstandlechner[1,2,3], Maria Laggner [1,2], Dragan Copic [1,2], Katharina Klas [1,2], Martin Direder[1,2], Yiyan Chen [4,5], Bahar Golabi[4], Werner Haslik [3], Christine Radtke [3], Erwin Tschachler [4], Konrad Hötzenecker[6], Hendrik Jan Ankersmit[1,2*], Michael Mildner [4*]

[1]Laboratory for Cardiac and Thoracic Diagnosis, Regeneration and Applied Immunology, Department of Thoracic Surgery, Medical University of Vienna

[2]Aposcience AG (FN 308089y), Dresdner Straße 87/A21, Vienna, Austria.

[3]Department of Plastic and Reconstructive Surgery, Medical University of Vienna, Vienna, Austria

[4]Department of Dermatology, Medical University of Vienna, Vienna, Austria

[5]University of Applied Sciences FH Campus Wien, Vienna, Austria

[6]Department of Thoracic Surgery, Medical University of Vienna, Vienna, Austria

* contributed equally to this work

corresponding authors:

Michael Mildner, PhD

Department of Dermatology, Medical University of Vienna

Lazarettgasse 14, 1090 Vienna, Austria

e-mail: michael.mildner@meduniwien.ac.at

phone: +43-1-40400-73507

fax: +43-1-40400-73590

	Sex	Ethnicity	Age	Location	Etiology
Skin 1	F	caucasian	30	abdomen	healthy skin
Skin 2	F	caucasian	36	abdomen	healthy skin
Skin 3	F	caucasian	43	abdomen	healthy skin
			**Mean 36+/-6,506**		
Scar 1	F	caucasian	75	forearm	injury
Scar 2	M	caucasian	24	calf	burn
Scar 3	F	caucasian	54	axilla	surgical scar
			**Mean 51±26.6**		
			**p=0.391**		

Table S1: Skin and scar donor demographics. Statistical significance of age was calculated using Student-t-test, p-value > 0.05, not significant.

**Target**	**Supplier**	**Product Nr.**	**Host species**	**Dilution**	**Application**
DPP4	abcam	ab215711	rabbit monoclonal	1:1000	IF (FFPE), WB
PLAU	Novus biologicals	NBP2-20819	rabbit polyclonal	1:100	IF (FFPE), WB
αSMA	abcam	ab7817	mouse monclonal	1:200	WB
Fibronectin	abcam	ab2413	rabbit polyclonal	1:500	IF (FFPE)
COL3a1	abcam	ab7778	rabbit polyclonal	1:200	IF
GAPDH	abcam	ab8245	mouse monclonal	1:10000	WB
SMAD 2/3	Cell signaling	Rabbit mAb 8685	rabbit monoclonal	1:500	WB
pSMAD2	Cell signaling	mAb 5339	rabbit polyclonal	1:1000	WB
pSMAD1/5/9	Cell signaling	mAb 13820	rabbit polyclonal	1:1000	WB
ERK 1/2	Cell signaling	Rabbit mAb #[illegible]	rabbit polyclonal	1:1000	WB
pERK1/2	Cell signaling	mAb 4376	rabbit polyclonal	1:1000	WB
Alexa fluor® 546 anti-mouse IgG (H +	Invitrogen	A-11030	goat polyclonal	1:500	IF, 2nd step
Alexa fluor® 546 anti-rabbit IgG (H + L)	Invitrogen	A-11035	goat polyclonal	1:500	IF, 2nd step
Anti-mouse, HRP-conjugated	GE Healthcare	GENX-A931	goat polyclonal	1:10 000	WB, 2nd step
Anti-rabbit, HRP-conjugated	Bio-Rad	#1706515	goat polyclonal	1:10 000	WB, 2nd step

Table S2: Antibodies used in experiments

Gene	Forward primer	Reverse Primer
B2M	5'-GATGAGTATGCCTGCCGTGTG-3'	5'- CAATCCAAATGCGGCATCT-3'
PLAU	5'-UAAUUCUUCUGGAGGAGAGGAGGGC-3'	5'-GCCCUCCUCUCCUCCAGAAGAAUUA-3'
DPP4	5'GGAAUGCCAGGAGGAAGGAAUCUUU-3'	5'-AAAGAUUCCUUCCUCCUGGCAUUCC-3'
COL1A1	5'-GTGCTAAAGGTGCCAATGGT-3'	5'-CTCCTCGCTTTCCTTCCTCT-3'
COL3A1	5'-GTCCATGGATGGTGGTTTTC-3'	5'-CACCTTCATTTGACCCCATC-3'
COL5A1	5'-GTCCATACCCGCTGGAAA-3'	5'-TCCATCAGGCAAGTTGTGAA-3
FN1	5'-CTGCAGCCACAACTTCTCTG-3'	5'-AGTTGCCACCAAGTTTGCTT-3'

Table S3: Primers used in PCR analyses

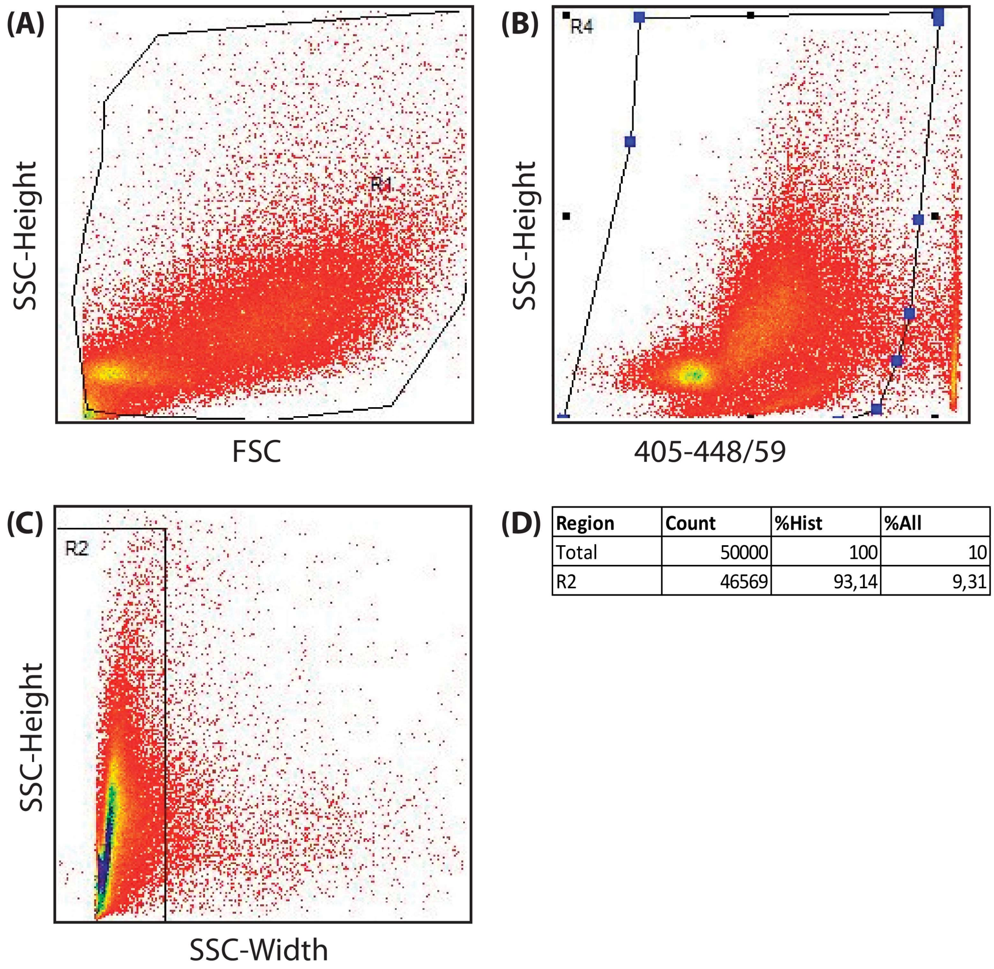

Region	Count	%Hist	%All
Total	50000	100	10
R2	46569	93,14	9,31

Figure S1: FACS gating strategy for DAPI-negative cell sorting

A) Cells from whole human or mouse skin, gated in side scatter (SSC) and forward scatter (FSC) to include all celltypes. B) Gating to exclude DAPI-positive cells C) Exclusion of cell doublets D) percentage and cell count per gating

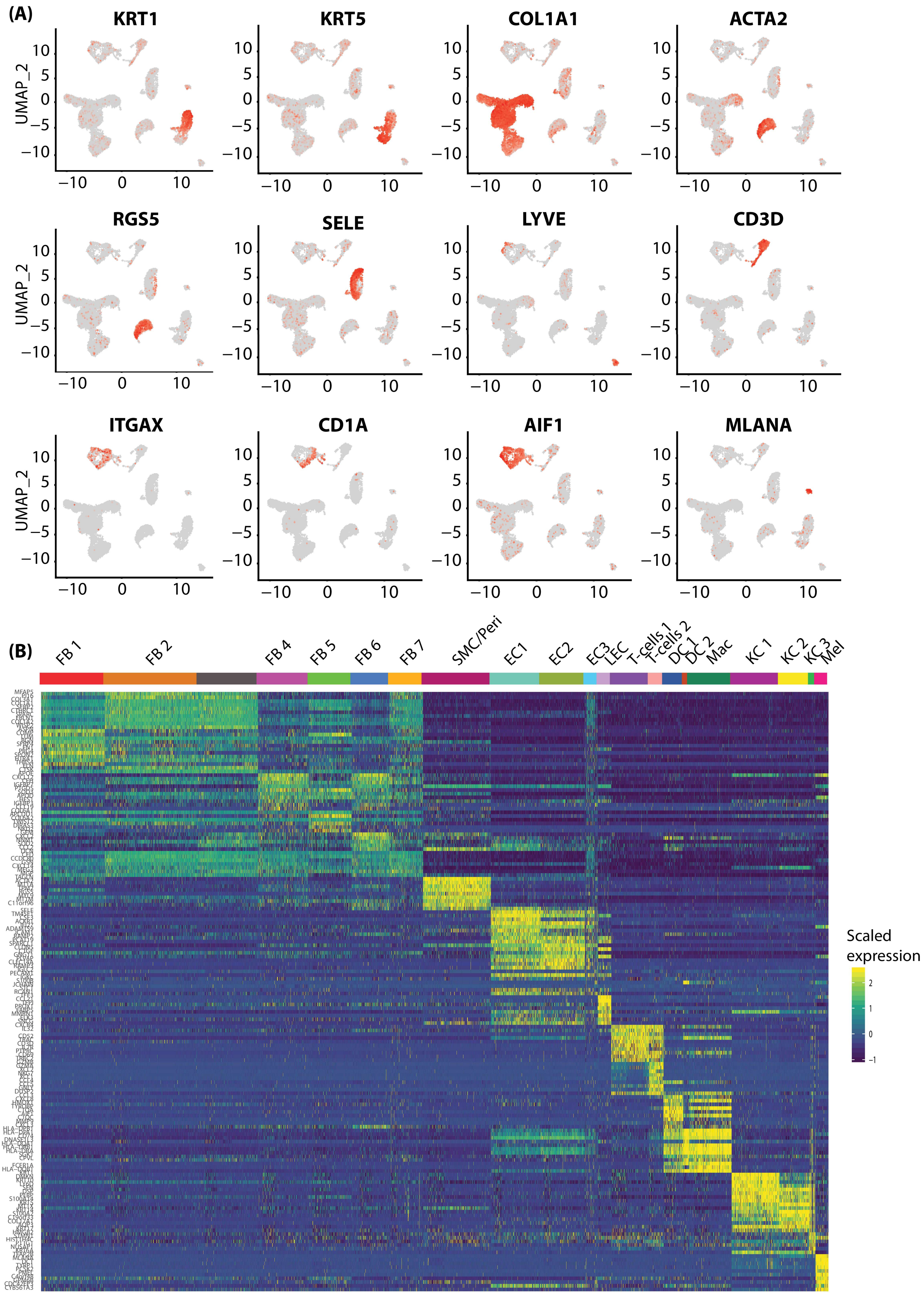
(A)
KRT1
KRT5
COL1A1
ACTA2
RGS5
SELE
LYVE
CD3D
ITGAX
CD1A
AIF1
MLANA
UMAP_2
(B)
FB 1
FB 2
FB 4
FB 5
FB 6
FB 7
SMC/Peri
EC1
EC2
EC3
LEC
T-cells 1
T-cells 2
DC 1
DC 2
Mac
KC 1
KC 2
KC 3
Mel
Scaled expression

Figure S2: Identification of cell types by marker genes and marker gene expression patterns in human skin and scar.

A) Feature Plots of cluster markers *KRT1* (Keratin1) for spinous and granular keratinocytes (KCs), *KRT5* (Keratin 5) for basal KCs, *COL1A1* (collagen I alpha 1) for ibroblasts (FBs), *ACTA2* (smooth muscle actin) for smooth muscle cells and myofibroblasts, *RGS5* (Regulator Of G Protein Signaling 5) for pericytes, *SELE* (E-selectin) for endothelial cells, *LYVE1* (Lymphatic Vessel Endothelial Hyaluronan Receptor 1) for lymphatic endothelial cells, *CD3D* (cluster of differentiation 3D) for Tcells, *ITGAX* (Integrin Subunit Alpha X, CD11C) and *CD1A* for dendritic cells, *AIF1* (Allograft Inflammatory Factor 1) for macrophages, and *MLANA* (Melan-A) for melanocytes. In feature plots, normalized log expression of the respective gene is mapped onto the UMAP-plot. B) Heatmap of top 10 clustermarker (upregulated genes of each cluster compared to the rest of the dataset). Heatmap shows scaled expression values for genes, rows represent genes, columns represent individual cells. DEGs were calculated per cluster comparing scar versus skin using Wilcoxon rank sum test, including genes with average logarithmic fold change (avglogFC) of > 0.1 or < -0.1 and Bonferroni-adjusted p-value <0.05. Feature plot shows projection of nDEG onto the UMAP-plot, color intensity represents nDEG. UMAP, uniform manifold approximation and projection.

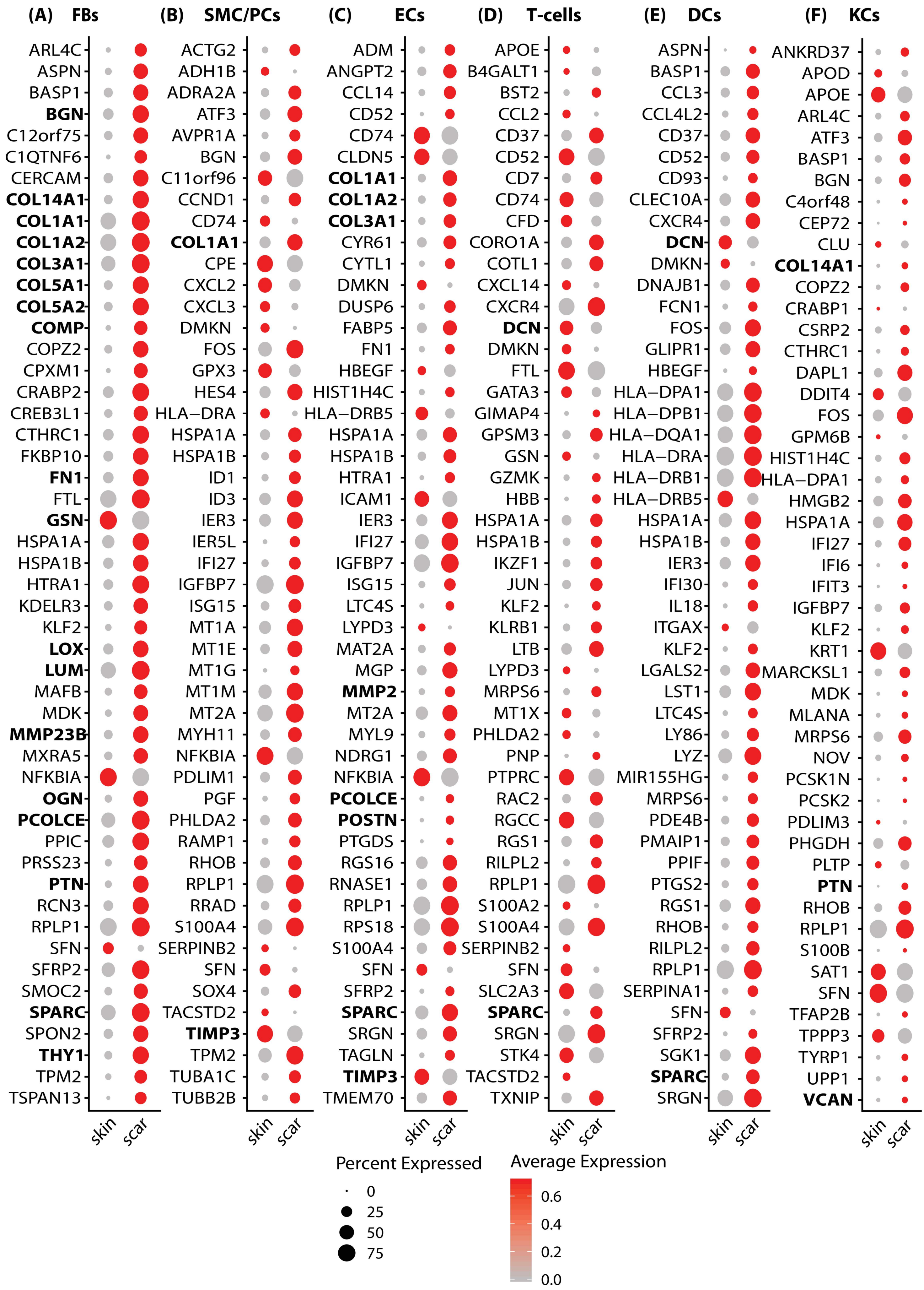
(A) FBs
ARL4C
ASPN
BASP1
BGN
C12orf75
C1QTNF6
CERCAM
COL14A1
COL1A1
COL1A2
COL3A1
COL5A1
COL5A2
COMP
COPZ2
CPXM1
CRABP2
CREB3L1
CTHRC1
FKBP10
FN1
FTL
GSN
HSPA1A
HSPA1B
HTRA1
KDELR3
KLF2
LOX
LUM
MAFB
MDK
MMP23B
MXRA5
NFKBIA
OGN
PCOLCE
PPIC
PRSS23
PTN
RCN3
RPLP1
SFN
SFRP2
SMOC2
SPARC
SPON2
THY1
TPM2
TSPAN13
skin
scar
(B) SMC/PCs
ACTG2
ADH1B
ADRA2A
ATF3
AVPR1A
BGN
C11orf96
CCND1
CD74
COL1A1
CPE
CXCL2
CXCL3
DMKN
FOS
GPX3
HES4
HLA–DRA
HSPA1A
HSPA1B
ID1
ID3
IER3
IER5L
IFI27
IGFBP7
ISG15
MT1A
MT1E
MT1G
MT1M
MT2A
MYH11
NFKBIA
PDLIM1
PGF
PHLDA2
RAMP1
RHOB
RPLP1
RRAD
S100A4
SERPINB2
SFN
SOX4
TACSTD2
TIMP3
TPM2
TUBA1C
TUBB2B
skin
scar
(C) ECs
ADM
ANGPT2
CCL14
CD52
CD74
CLDN5
COL1A1
COL1A2
COL3A1
CYR61
CYTL1
DMKN
DUSP6
FABP5
FN1
HBEGF
HIST1H4C
HLA–DRB5
HSPA1A
HSPA1B
HTRA1
ICAM1
IER3
IFI27
IGFBP7
ISG15
LTC4S
LYPD3
MAT2A
MGP
MMP2
MT2A
MYL9
NDRG1
NFKBIA
PCOLCE
POSTN
PTGDS
RGS16
RNASE1
RPLP1
RPS18
S100A4
SFN
SFRP2
SPARC
SRGN
TAGLN
TIMP3
TMEM70
skin
scar
(D) T-cells
APOE
B4GALT1
BST2
CCL2
CD37
CD52
CD7
CD74
CFD
CORO1A
COTL1
CXCL14
CXCR4
DCN
DMKN
FTL
GATA3
GIMAP4
GPSM3
GSN
GZMK
HBB
HSPA1A
HSPA1B
IKZF1
JUN
KLF2
KLRB1
LTB
LYPD3
MRPS6
MT1X
PHLDA2
PNP
PTPRC
RAC2
RGCC
RGS1
RILPL2
RPLP1
S100A2
S100A4
SERPINB2
SFN
SLC2A3
SPARC
SRGN
STK4
TACSTD2
TXNIP
skin
scar
(E) DCs
ASPN
BASP1
CCL3
CCL4L2
CD37
CD52
CD93
CLEC10A
CXCR4
DCN
DMKN
DNAJB1
FCN1
FOS
GLIPR1
HBEGF
HLA–DPA1
HLA–DPB1
HLA–DQA1
HLA–DRA
HLA–DRB1
HLA–DRB5
HSPA1A
HSPA1B
IER3
IFI30
IL18
ITGAX
KLF2
LGALS2
LST1
LTC4S
LY86
LYZ
MIR155HG
MRPS6
PDE4B
PMAIP1
PPIF
PTGS2
RGS1
RHOB
RILPL2
RPLP1
SERPINA1
SFN
SFRP2
SGK1
SPARC
SRGN
skin
scar
(F) KCs
ANKRD37
APOD
APOE
ARL4C
ATF3
BASP1
BGN
C4orf48
CEP72
CLU
COL14A1
COPZ2
CRABP1
CSRP2
CTHRC1
DAPL1
DDIT4
FOS
GPM6B
HIST1H4C
HLA–DPA1
HMGB2
HSPA1A
IFI27
IFI6
IFIT3
IGFBP7
KLF2
KRT1
MARCKSL1
MDK
MLANA
MRPS6
NOV
PCSK1N
PCSK2
PDLIM3
PHGDH
PLTP
PTN
RHOB
RPLP1
S100B
SAT1
SFN
TFAP2B
TPPP3
TYRP1
UPP1
VCAN
skin
scar
Percent Expressed
0
25
50
75
Average Expression
0.6
0.4
0.2
0.0

Figure S3: Top 50 regulated genes per cell group in scar compared to skin.

In cell groups, i.e. A) Fibroblasts (FBs), B) smooth muscle cells and pericytes (SMC/PCs), C) endothelial cells (ECs), D) T-cells, E) dendritic cells (DCs), and keratinocytes (KCs), differentially expressed genes (DEGs) were calculated comparing scar versus skin using Wilcoxon rank sum test, including genes with average logarithmic fold change (avglogFC) of > 0.1 or < -0.1 and Bonferroni-adjusted p-value <0.05. For each cell group, top 50 DEGs according to lowest adjusted p-value are displayed, split by skin and scar. ECM-related genes are in bold font. Dot size represents percent of cells expressing the respective gene, color correlates with average expression.

Figure S3

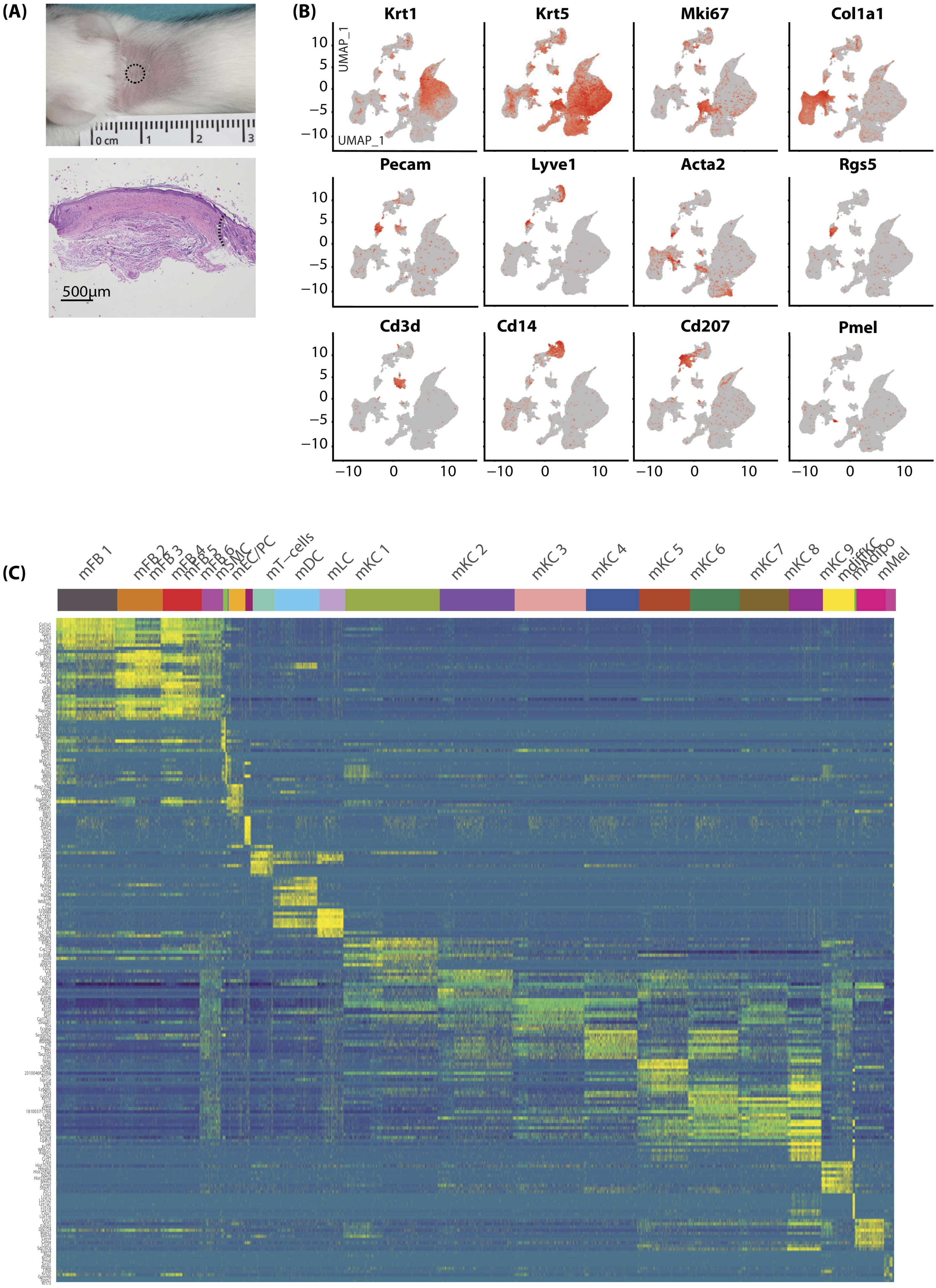

(A)
0 cm
1
2
3
500µm
(B)
Krt1
Krt5
Mki67
Col1a1
Pecam
Lyve1
Acta2
Rgs5
Cd3d
Cd14
Cd207
Pmel
UMAP_1
−10
0
10
5
−5
(C)
mFB 1
mFB 2
mFB 3
mFB 4
mFB 5
mFB 6
mSMC
mEC/PC
mT−cells
mDC
mLC
mKC 1
mKC 2
mKC 3
mKC 4
mKC 5
mKC 6
mKC 7
mKC 8
mKC 9
mdiffKC
mAdipo
mMel

Figure S4: Identification of cell types by marker genes and gene expression patterns in mouse scars.

A) Macroscopic image and 4x microscopic image illustrating scar size relations. Dotted circle and dotted lines indicate scar tissue. B) Feature Plots of cluster markers *Krt1* (Keratin1) for spinous and granular keratinocytes (KCs), *Krt5* (Keratin 5) for basal KCs, *Mki67* (Marker Of Proliferation Ki-67) for proliferating cells, *Col1a1* (collagen I alpha 1) for fibroblasts, *Pecam* (Platelet And Endothelial Cell Adhesion Molecule 1) for endothelial cells, *Lyve1* (Lymphatic Vessel Endothelial Hyaluronan Receptor 1) for lymphatic endothelial cells, *Acta2* (smooth muscle actin) for smooth muscle cells and myofibroblasts, *Rgs5* (Regulator Of G Protein Signaling 5) for pericytes, *Cd3d* (cluster of differentiation 3D) for T-cells, *Cd14* for dendritic cells, *Cd207* (Langerin) for Langerhans cells, and *Pmel* (Premelanosome Protein) for melanocytes. In feature plots, normalized log expression of the respective gene is mapped onto the UMAP-plot. C) heatmap of top 10 upregulated clustermarker (differentially expressed genes of each cluster compared to the rest of the dataset). Heatmap showing scaled expression values for genes, rows represent genes, columns represent individual cells.

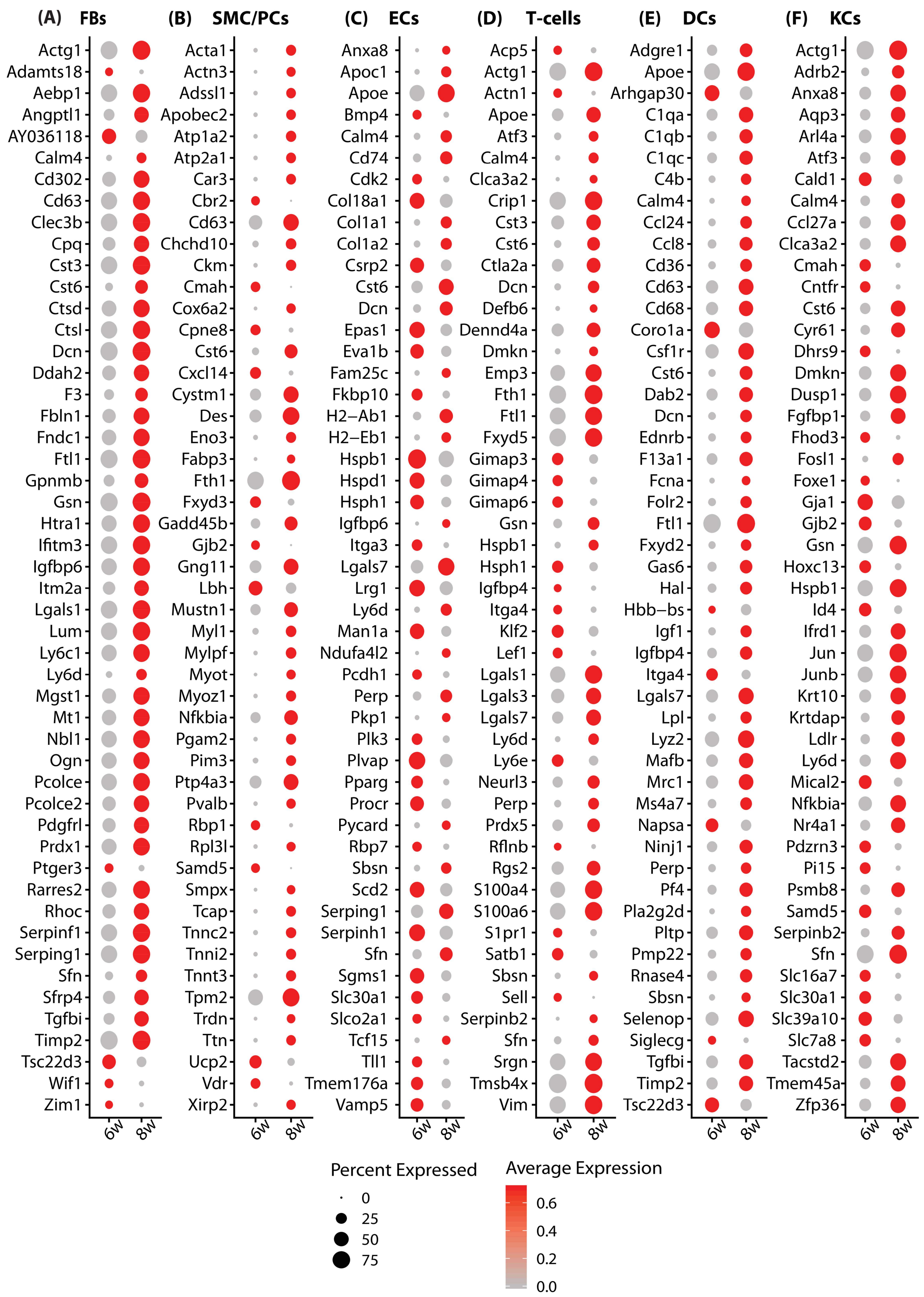
(A) FBs
Actg1
Adamts18
Aebp1
Angptl1
AY036118
Calm4
Cd302
Cd63
Clec3b
Cpq
Cst3
Cst6
Ctsd
Ctsl
Dcn
Ddah2
F3
Fbln1
Fndc1
Ftl1
Gpnmb
Gsn
Htra1
Ifitm3
Igfbp6
Itm2a
Lgals1
Lum
Ly6c1
Ly6d
Mgst1
Mt1
Nbl1
Ogn
Pcolce
Pcolce2
Pdgfrl
Prdx1
Ptger3
Rarres2
Rhoc
Serpinf1
Serping1
Sfn
Sfrp4
Tgfbi
Timp2
Tsc22d3
Wif1
Zim1
6W
8W
(B) SMC/PCs
Acta1
Actn3
Adssl1
Apobec2
Atp1a2
Atp2a1
Car3
Cbr2
Cd63
Chchd10
Ckm
Cmah
Cox6a2
Cpne8
Cst6
Cxcl14
Cystm1
Des
Eno3
Fabp3
Fth1
Fxyd3
Gadd45b
Gjb2
Gng11
Lbh
Mustn1
Myl1
Mylpf
Myot
Myoz1
Nfkbia
Pgam2
Pim3
Ptp4a3
Pvalb
Rbp1
Rpl3l
Samd5
Smpx
Tcap
Tnnc2
Tnni2
Tnnt3
Tpm2
Trdn
Ttn
Ucp2
Vdr
Xirp2
6W
8W
(C) ECs
Anxa8
Apoc1
Apoe
Bmp4
Calm4
Cd74
Cdk2
Col18a1
Col1a1
Col1a2
Csrp2
Cst6
Dcn
Epas1
Eva1b
Fam25c
Fkbp10
H2−Ab1
H2−Eb1
Hspb1
Hspd1
Hsph1
Igfbp6
Itga3
Lgals7
Lrg1
Ly6d
Man1a
Ndufa4l2
Pcdh1
Perp
Pkp1
Plk3
Plvap
Pparg
Procr
Pycard
Rbp7
Sbsn
Scd2
Serping1
Serpinh1
Sfn
Sgms1
Slc30a1
Slco2a1
Tcf15
Tll1
Tmem176a
Vamp5
6W
8W
(D) T-cells
Acp5
Actg1
Actn1
Apoe
Atf3
Calm4
Clca3a2
Crip1
Cst3
Cst6
Ctla2a
Dcn
Defb6
Dennd4a
Dmkn
Emp3
Fth1
Ftl1
Fxyd5
Gimap3
Gimap4
Gimap6
Gsn
Hspb1
Hsph1
Igfbp4
Itga4
Klf2
Lef1
Lgals1
Lgals3
Lgals7
Ly6d
Ly6e
Neurl3
Perp
Prdx5
Rflnb
Rgs2
S100a4
S100a6
S1pr1
Satb1
Sbsn
Sell
Serpinb2
Sfn
Srgn
Tmsb4x
Vim
6W
8W
(E) DCs
Adgre1
Apoe
Arhgap30
C1qa
C1qb
C1qc
C4b
Calm4
Ccl24
Ccl8
Cd36
Cd63
Cd68
Coro1a
Csf1r
Cst6
Dab2
Dcn
Ednrb
F13a1
Fcna
Folr2
Ftl1
Fxyd2
Gas6
Hal
Hbb−bs
Igf1
Igfbp4
Itga4
Lgals7
Lpl
Lyz2
Mafb
Mrc1
Ms4a7
Napsa
Ninj1
Perp
Pf4
Pla2g2d
Pltp
Pmp22
Rnase4
Sbsn
Selenop
Siglecg
Tgfbi
Timp2
Tsc22d3
6W
8W
(F) KCs
Actg1
Adrb2
Anxa8
Aqp3
Arl4a
Atf3
Cald1
Calm4
Ccl27a
Clca3a2
Cmah
Cntfr
Cst6
Cyr61
Dhrs9
Dmkn
Dusp1
Fgfbp1
Fhod3
Fosl1
Foxe1
Gja1
Gjb2
Gsn
Hoxc13
Hspb1
Id4
Ifrd1
Jun
Junb
Krt10
Krtdap
Ldlr
Ly6d
Mical2
Nfkbia
Nr4a1
Pdzrn3
Pi15
Psmb8
Samd5
Serpinb2
Sfn
Slc16a7
Slc30a1
Slc39a10
Slc7a8
Tacstd2
Tmem45a
Zfp36
6W
8W
Percent Expressed
0
25
50
75
Average Expression
0.6
0.4
0.2
0.0

Figure S5: Top 50 regulated genes per cell group in 6 weeks and 8 weeks old murine scars.

In A) Fibroblasts (FBs), B) smooth muscle cells and pericytes (SMC/PCs), C) endothelial cells (ECs), D) T-cells, E) dendritic cells (DCs), and keratinocytes (KCs), differentially expressed genes (DEGs) were calculated comparing 8 weeks versus 6 weeks old mouse scars using Wilcoxon rank sum test, including genes with average logarithmic fold change (avg_logFC) of >0.1 or <-0.1 and Bonferroni-adjusted p-value <0.05. For each cell group, top 50 DEGs according to lowest adjusted p-value are displayed, split by timepoint. Dot size represents percent of cells expressing the respective gene, color correlates with average expression.

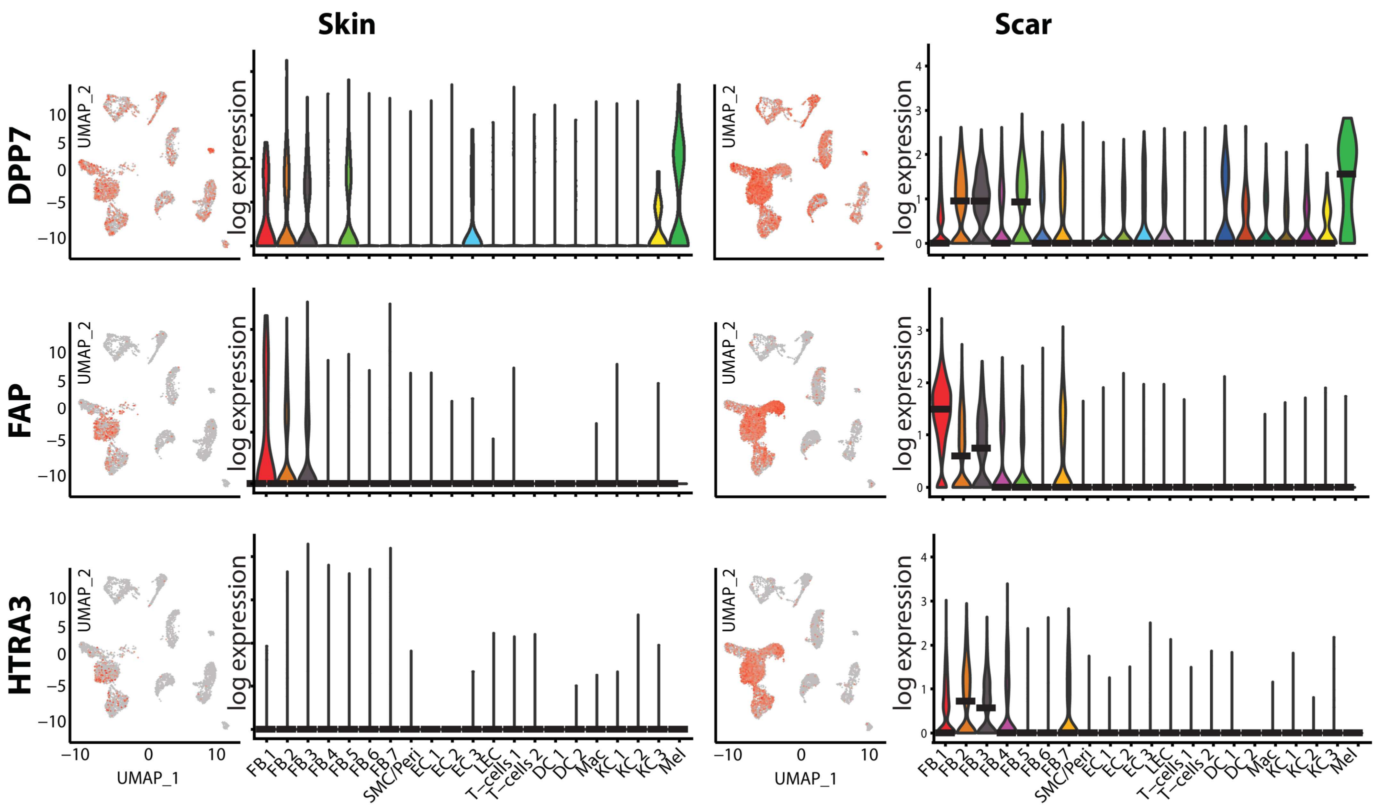

Figure S6: Feature Plots and Violin plots of serine proteases.

A-C) Feature plots and violin plots of serine proteases in human skin and scar. *DPP7* (dipeptidylpeptidase 7), *FAP* (Fibroblast Activation Protein Alpha), *HTRA3* (High-Temperature Requirement A Serine Peptidase 3). In violin plots, dots represent individual cells, y-axis represents log2 fold change of the normalized genes and log-transformed single cell expression. Vertical lines in violin plots represent maximum expression, shape of each violin represents all results, and width of each violin represents frequency of respective expression level. In feature plots, normalized log expression of the respective gene is mapped onto the UMAP-plot. Color intensity indicates level of gene expressions. UMAP, uniform manifold approximation and projection

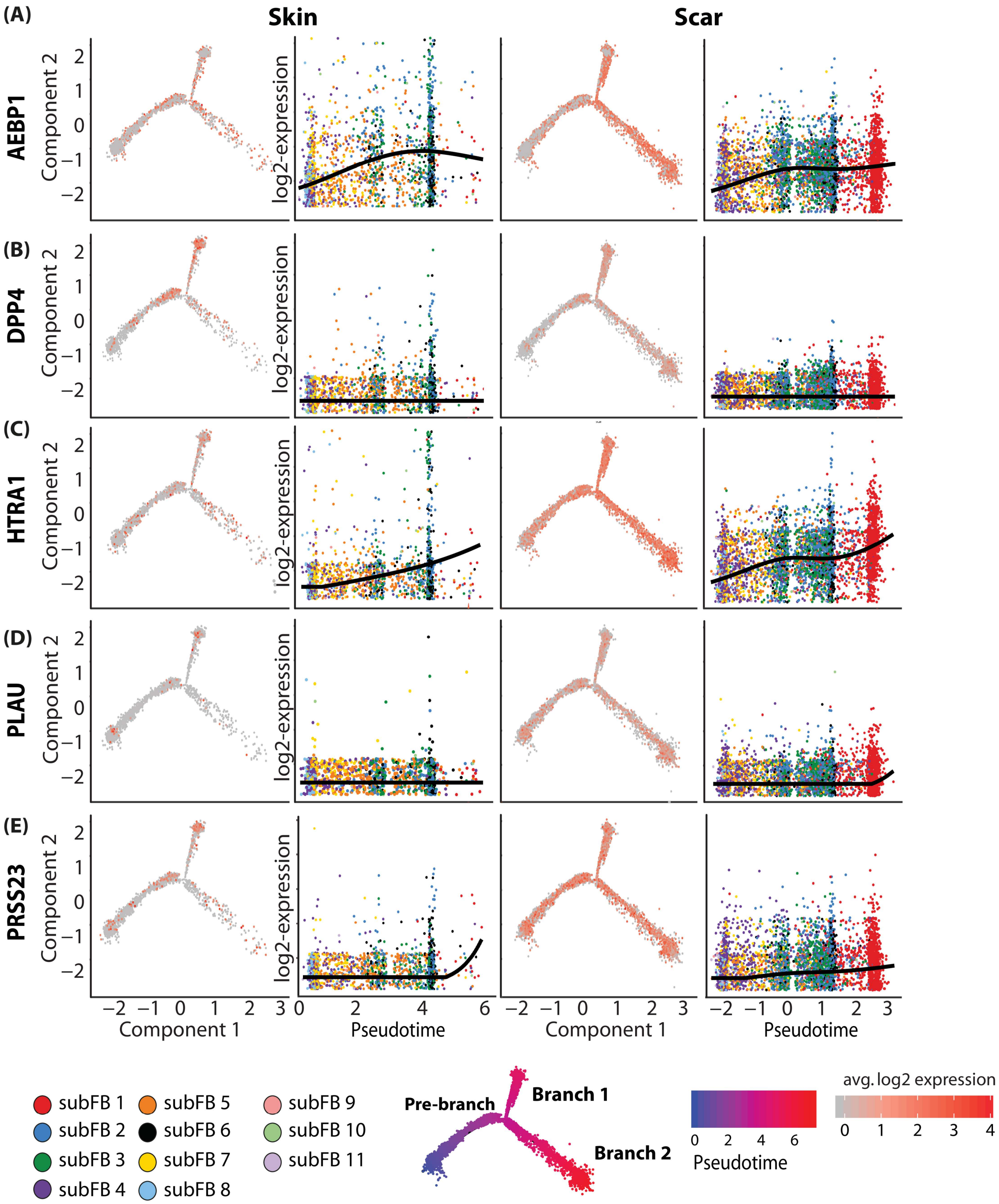
(A)
(B)
(C)
(D)
(E)
Skin
Scar
AEBP1
DPP4
HTRA1
PLAU
PRSS23
Component 2
log2-expression
Component 1
Pseudotime
subFB 1
subFB 2
subFB 3
subFB 4
subFB 5
subFB 6
subFB 7
subFB 8
subFB 9
subFB 10
subFB 11
Pre-branch
Branch 1
Branch 2
Pseudotime
avg. log2 expression

Figure S7: Pseudotime analysis corroborates the putative role of serine proteases as drivers of scar maturation.

A-E) Trajectory plots and pseudotime plots of Serine proteases in skin and scar FBs. *AEBP1* (adipocyte enhancer binding protein 1), *DPP4* (dipeptidyl-peptidase 4), *HTRA1* (High-Temperature Requirement A Serine Peptidase 1), *PLAU* (urokinase), *PRSS23* (Serine protease 23). In trajectory plots, normalized log expressions are plotted on the trajectories, split by skin and scar. In pseudotime plots, normalized log expressions are plotted against the pseudotime axis, and a spline curve represents expression dynamics over pseudotime. Y-axis, normalized log expression of respective gene, x-axis, pseudotime.

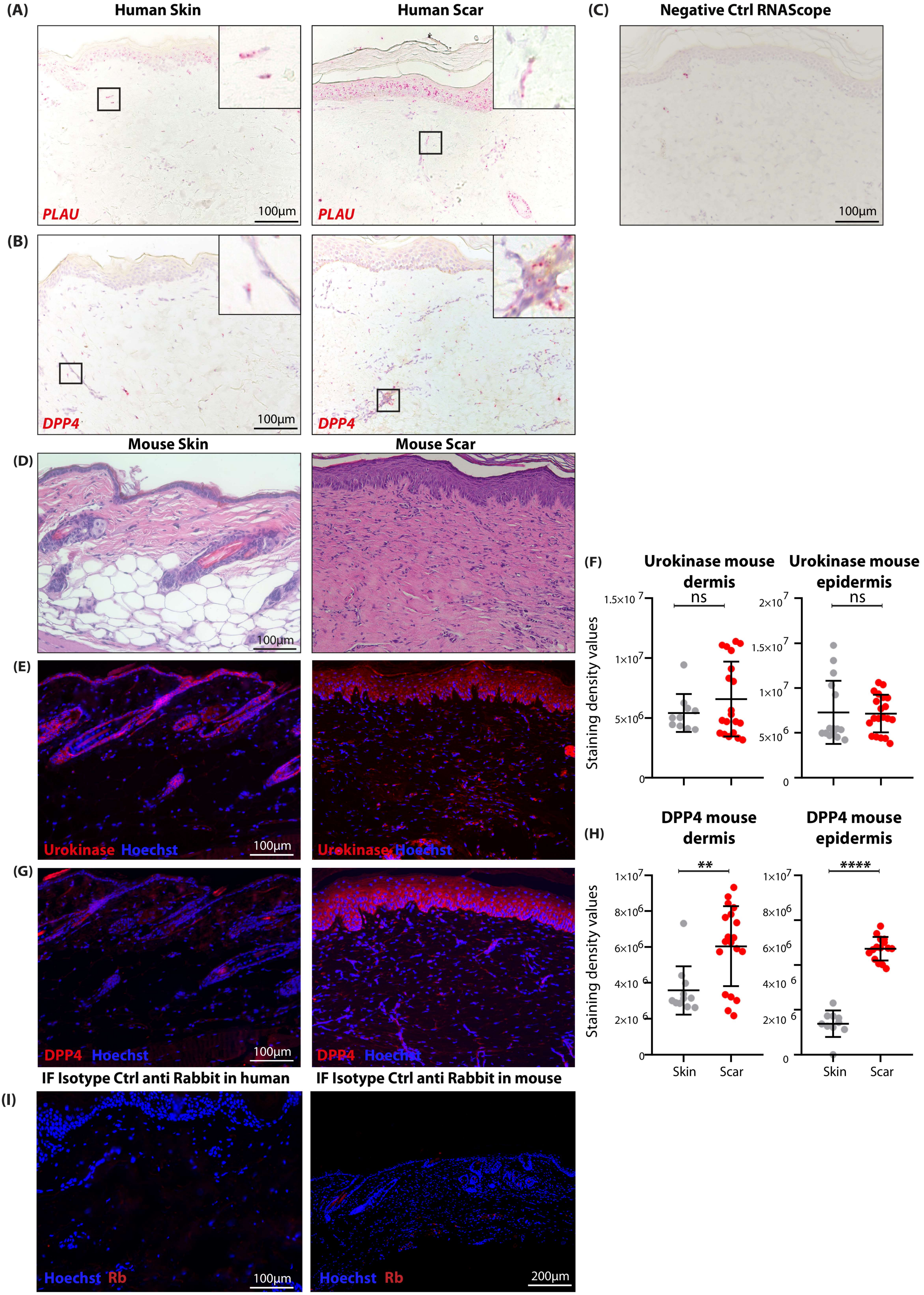

(A)
Human Skin
Human Scar
(C)
Negative Ctrl RNAScope
PLAU
100µm
PLAU
100µm
(B)
DPP4
100µm
DPP4
Mouse Skin
Mouse Scar
(D)
100µm
(E)
Urokinase Hoechst
100µm
Urokinase Hoechst
(F)
Urokinase mouse dermis
Urokinase mouse epidermis
ns
ns
Staining density values
1.5×10 7
1×10 7
5×10 6
0
2×10 7
1.5×10 7
1×10 7
5×10 6
0
(G)
DPP4 Hoechst
100µm
DPP4 Hoechst
(H)
DPP4 mouse dermis
DPP4 mouse epidermis
**
****
1×10 7
8×10 6
6×10 6
4×10 6
2×10 6
0
Skin
Scar
IF Isotype Ctrl anti Rabbit in human
IF Isotype Ctrl anti Rabbit in mouse
(I)
Hoechst Rb
100µm
Hoechst Rb
200µm

Figure S8: In-situ hybridization of *PLAU* and *DPP4* in human skin and scar.

A, B) RNAScope in-hybridization of skin and scar tissue with PLAU- and DPP4-probes. Red dots indicate single mRNA molecules. Inserts show high magnification micrographs. C) Negative control image of RNAScope experiments. Universal negative control with probes targeting the DapB gene (accession # EF191515) from the Bacillus subtilis strain SMY was used. D) H&E images from mouse skin and scar. E, G) Immunofluorescence stainings from E) urokinase and G) DPP4 in mouse skin and scars. F,H) Quantification of staining intensity from urokinase and DPP4 in skin and scar, analyzed separately for epidermis and dermis. Urokinase mouse dermis $p=0.2813$; Urokinase mouse epidermis $p=0.8915$; DPP4 mouse dermis $p=0.0025$; DPP4 mouse epidermis $p<0.0001$ I) Isotype ctrl images of Rb IgG in human and mouse samples. $n=3$ biologically independent samples of healthy mouse skin and $n=4$ samples of mouse scars were analyzed. Lines and error bars represent mean and standard deviation. From each sample, five regions of interest per sample were quantified. For ELISA, analysis was performed in duplicates for three donors each. Statistical significance was tested using two-tailed unpaired Student t-test. NS $p>0.05$, *$p<0.05$, **$p<0.01$, ***$p<0.001$.

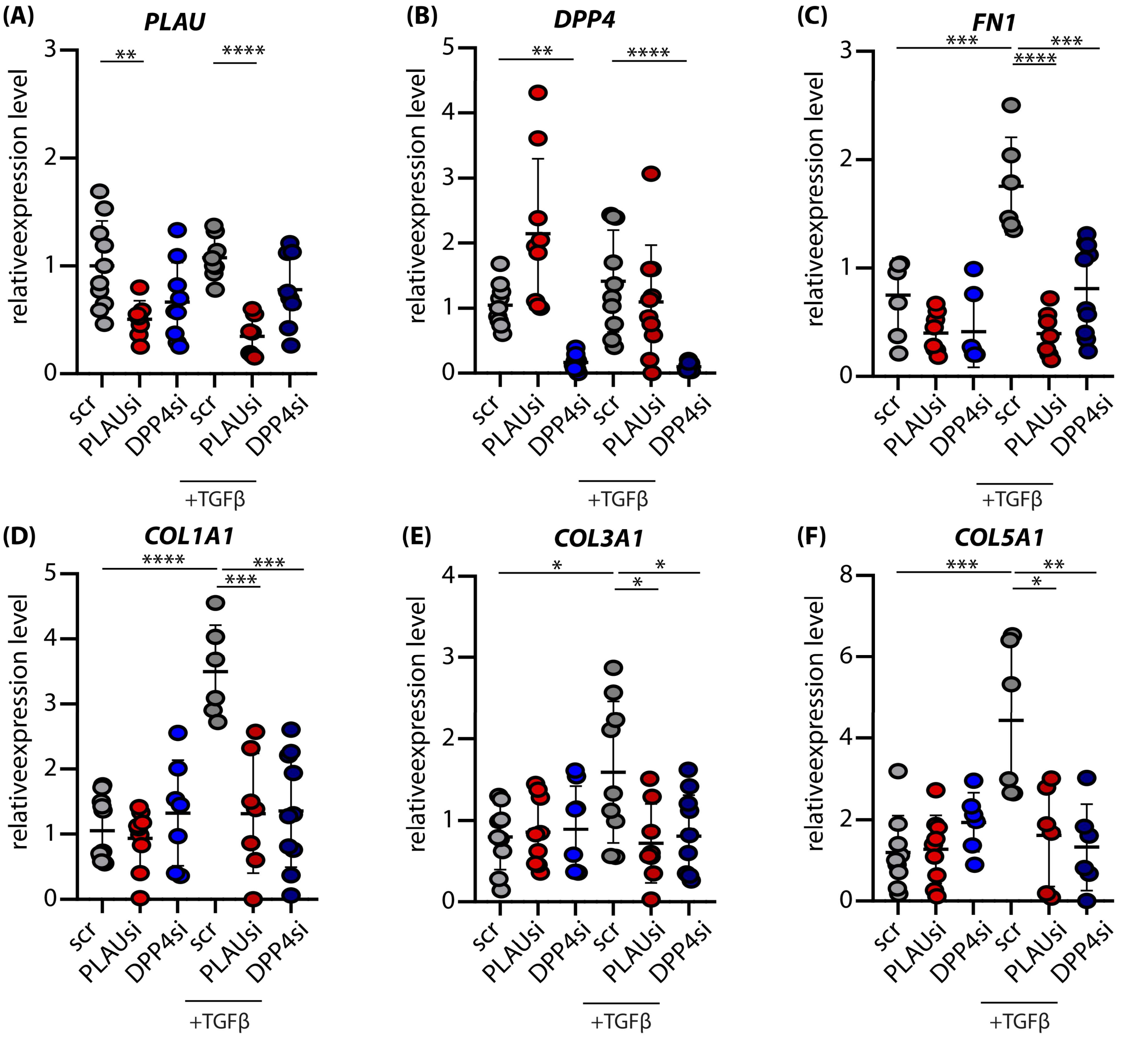

Figure S9: PCR analysis of serine protease and ECM genes after knockdown.

PCR analysis of *PLAU*, *DPP4*, *FN1*, *COL1A1*, *COL3A1* and *COL5A1* expression in primary FBs after stimulation with scrambled (scr) RNA, small interfering (si) RNA targeting *PLAU* or *DPP4*, and upon stimulation with or without TGF_1. Y-axis represents fold expression as calculated in reference to housekeeping gene *B2M*. A) scr vs PLAUsi p=0.0249, scr vs PLAUsi + TGFβ1 p= p>0.001; B) scr + TGFβ1 vs DPP4si p=0.0012, scr + TGFβ1vs DPP4si + TGFβ1 p= p<0.0001 C) scr vs scr+ TGFβ1 p=0.0008, scr + TGFβ1 vs PLAUsi + TGFβ1 p<0.0001; scr + TGFβ1 vs DPP4si + TGFβ1=0.0008 D) scr vs scr+ TGFβ1 p= 0.0001, scr + TGFβ1 vs PLAUsi + TGFβ1 p= 0.0232; scr+ TGFβ1 vs DPPsi+ TGFβ1 p=0.0039 E) scr vs scr + TGFβ1 p= 0.242, scr vs PLAUsi+ TGFβ1 p=0.010, scr vs DPP4si+ TGFβ1 p=0.0291; F) scr vs scr+ TGFβ1 p= 0-0051, scr + TGFβ1 vs PLAUsi + TGFβ1 p= 0.0144; scr+ TGFβ1 vs DPPsi+ TGFβ1 p=0.040 n=6 biologically independent samples were analyzed in technical duplicates. Lines and error bars represent mean with standard deviation. Statistical significance was tested using one-way ANOVA with Tukey post-test. *p<0.05, **p<0.01, ***p<0.001, ****p<0.0001.

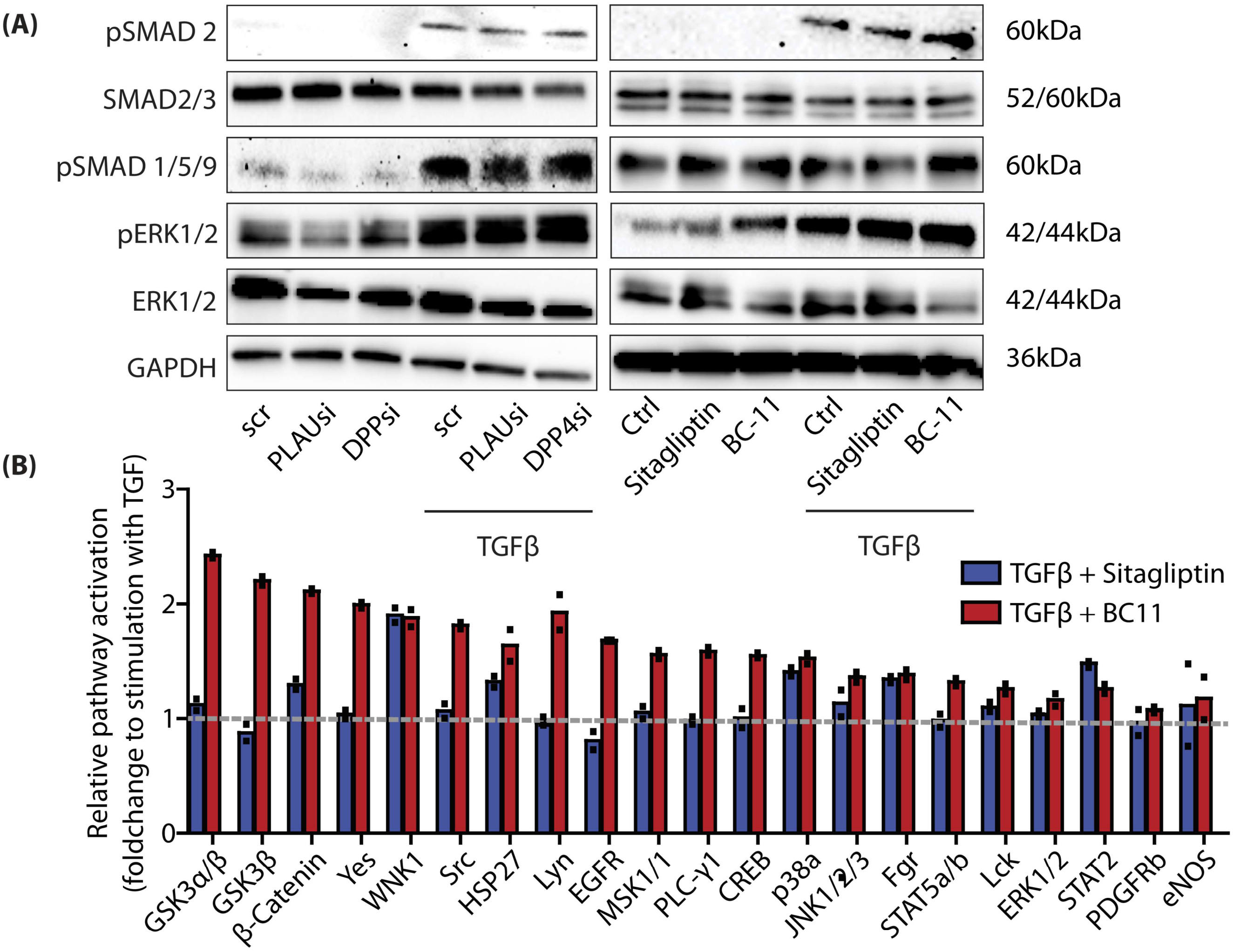

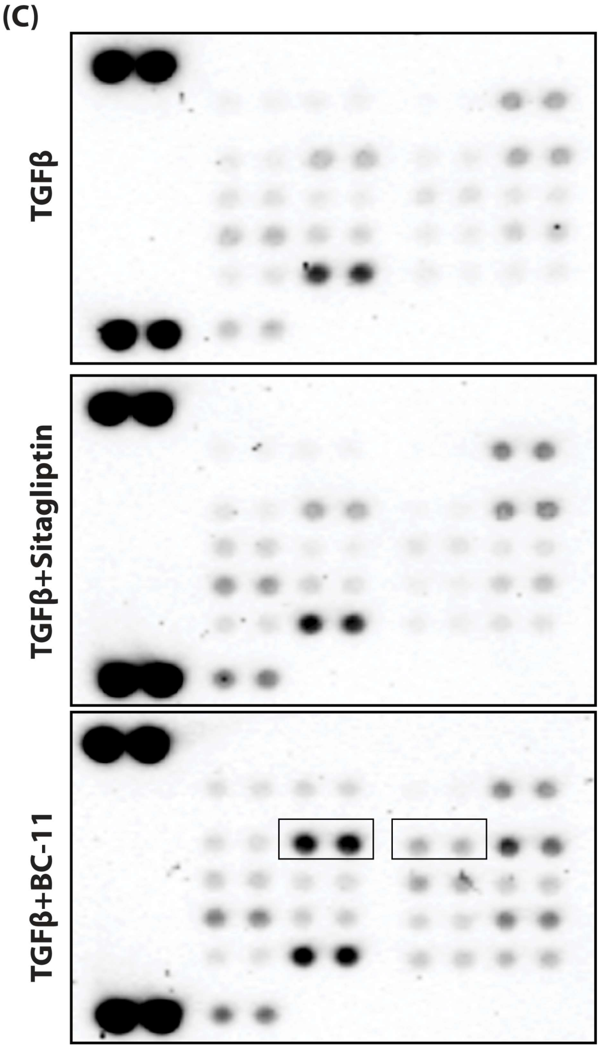

(D)

A1,2	A3,4	A5,6	A7,8	A9,10
Reference spots				
B1,2	B3,4	B5,6	B7,8	B9,10
	CREB	EGFR	eNOS	ERK1/2
C1,2	C3,4	C5,6	C7,8	C9,10
	Fgr	GSK3α/β	GSK3β	HSP27
D1,2	D3,4	D5,6	D7,8	D9,10
	JNK1/2/3	Lck	Lyn	MSK1/2
E1,2	E3,4	E5,6	E7,8	E9,10
	p38a	PDGF β	1 γ C	Src P
F1,2	F3,4	F5,6	F7,8	F9,10
	STAT2	STAT5a/b	WNK1	Yes
G1,2	G3,4	G5,6	G7,8	G9,10
Reference spots				Negative control (PBS)

Figure S10: Signaling pathway activation after TGFβ-stimulation and DPP4/PLAU inhibition.

A) Western blot of primary FB lysates stimulated with active TGFβ1 for 1h and analysis of canonical TGFβ1 signaling pathways. B) Analysis of non-canonical TGFβ1 signaling pathways and kinase pathways detected by proteome profiler from lysates of primary FBs after 1h stimulation with TGFβ1 alone, TGFβ1 with sitagliptin, and TGFβ1 with BC-11. Bars represent fold change compared to stimulation with TGFβ1 only, marked by dotted line. Proteome profiler analysis of signaling pathways of primary human skin FBs stimulated with TGFβ and inhibitors. Values show technical duplicates of pooled samples from n=3 biologically independent donors. C) TGFβ1, B) TGFβ1 and DPP4-inhibitor Sitagliptin, and TGFβ1 and PLAU-inhibitor BC-11. GSK3α/β and GSK3β are marked by squares in the BC-11 treated blot. D) Legend table for proteome profiler data points.

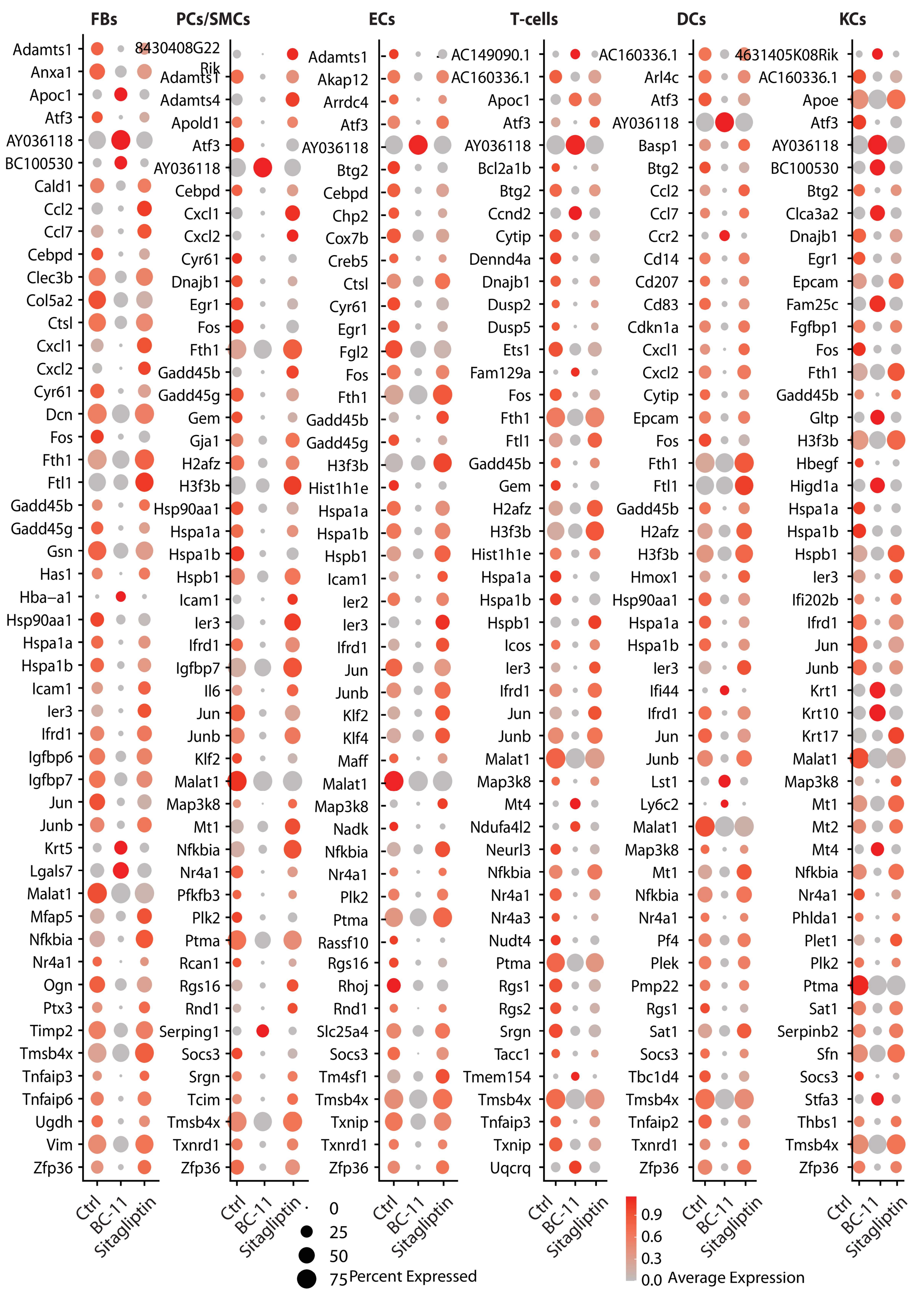

FBs
PCs/SMCs
ECs
T-cells
DCs
KCs
Adamts1
Anxa1
Apoc1
Atf3
AY036118
BC100530
Cald1
Ccl2
Ccl7
Cebpd
Clec3b
Col5a2
Ctsl
Cxcl1
Cxcl2
Cyr61
Dcn
Fos
Fth1
Ftl1
Gadd45b
Gadd45g
Gsn
Has1
Hba−a1
Hsp90aa1
Hspa1a
Hspa1b
Icam1
Ier3
Ifrd1
Igfbp6
Igfbp7
Jun
Junb
Krt5
Lgals7
Malat1
Mfap5
Nfkbia
Nr4a1
Ogn
Ptx3
Timp2
Tmsb4x
Tnfaip3
Tnfaip6
Ugdh
Vim
Zfp36
8430408G22Rik
Adamts1
Adamts4
Apold1
Atf3
AY036118
Cebpd
Cxcl1
Cxcl2
Cyr61
Dnajb1
Egr1
Fos
Fth1
Gadd45b
Gadd45g
Gem
Gja1
H2afz
H3f3b
Hsp90aa1
Hspa1a
Hspa1b
Hspb1
Icam1
Ier3
Ifrd1
Igfbp7
Il6
Jun
Junb
Klf2
Malat1
Map3k8
Mt1
Nfkbia
Nr4a1
Pfkfb3
Plk2
Ptma
Rcan1
Rgs16
Rnd1
Serping1
Socs3
Srgn
Tcim
Tmsb4x
Txnrd1
Zfp36
Adamts1
Akap12
Arrdc4
Atf3
AY036118
Btg2
Cebpd
Chp2
Cox7b
Creb5
Ctsl
Cyr61
Egr1
Fgl2
Fos
Fth1
Gadd45b
Gadd45g
H3f3b
Hist1h1e
Hspa1a
Hspa1b
Hspb1
Icam1
Ier2
Ier3
Ifrd1
Jun
Junb
Klf2
Klf4
Maff
Malat1
Map3k8
Nadk
Nfkbia
Nr4a1
Plk2
Ptma
Rassf10
Rgs16
Rhoj
Rnd1
Slc25a4
Socs3
Tm4sf1
Tmsb4x
Txnip
Txnrd1
Zfp36
AC149090.1
AC160336.1
Apoc1
Atf3
AY036118
Bcl2a1b
Btg2
Ccnd2
Cytip
Dennd4a
Dnajb1
Dusp2
Dusp5
Ets1
Fam129a
Fos
Fth1
Ftl1
Gadd45b
Gem
H2afz
H3f3b
Hist1h1e
Hspa1a
Hspa1b
Hspb1
Icos
Ier3
Ifrd1
Jun
Junb
Malat1
Map3k8
Mt4
Ndufa4l2
Neurl3
Nfkbia
Nr4a1
Nr4a3
Nudt4
Ptma
Rgs1
Rgs2
Srgn
Tacc1
Tmem154
Tmsb4x
Tnfaip3
Txnip
Uqcrq
AC160336.1
Arl4c
Atf3
AY036118
Basp1
Btg2
Ccl2
Ccl7
Ccr2
Cd14
Cd207
Cd83
Cdkn1a
Cxcl1
Cxcl2
Cytip
Epcam
Fos
Fth1
Ftl1
Gadd45b
H2afz
H3f3b
Hmox1
Hsp90aa1
Hspa1a
Hspa1b
Ier3
Ifi44
Ifrd1
Jun
Junb
Lst1
Ly6c2
Malat1
Map3k8
Mt1
Nfkbia
Nr4a1
Pf4
Plek
Pmp22
Rgs1
Sat1
Socs3
Tbc1d4
Tmsb4x
Tnfaip2
Txnrd1
Zfp36
4631405K08Rik
AC160336.1
Apoe
Atf3
AY036118
BC100530
Btg2
Clca3a2
Dnajb1
Egr1
Epcam
Fam25c
Fgfbp1
Fos
Fth1
Gadd45b
Gltp
H3f3b
Hbegf
Higd1a
Hspa1a
Hspa1b
Hspb1
Ier3
Ifi202b
Ifrd1
Jun
Junb
Krt1
Krt10
Krt17
Malat1
Map3k8
Mt1
Mt2
Mt4
Nfkbia
Nr4a1
Phlda1
Plet1
Plk2
Ptma
Sat1
Serpinb2
Sfn
Socs3
Stfa3
Thbs1
Tmsb4x
Zfp36
Ctrl
BC-11
Sitagliptin
0
25
50
75
Percent Expressed
0.9
0.6
0.3
0.0
Average Expression

Figure S11: Top 50 regulated genes per cell group in protease-inhibitor stimulated murine scars.

In A) fibroblasts (FBs), B) smooth muscle cells and pericytes (SMC/PCs), C) endothelial cells (ECs), D) T-cells, E) dendritic cells (DCs), and keratinocytes (KCs), differentially expressed genes (DEGs) were calculated comparing BC-11 and Sitagliptin-treated scars with Ctrl scars using Wilcoxon rank sum test, including genes with average logarithmic fold change (avglogFC) of > 0.1 or < -0.1 and Bonferroni-adjusted p-value <0.05. For each cell group, top 50 DEGs according to lowest adjusted p-value are displayed, split by timepoint. Dot size represents percent of cells expressing the respective gene, color correlates with average expression.

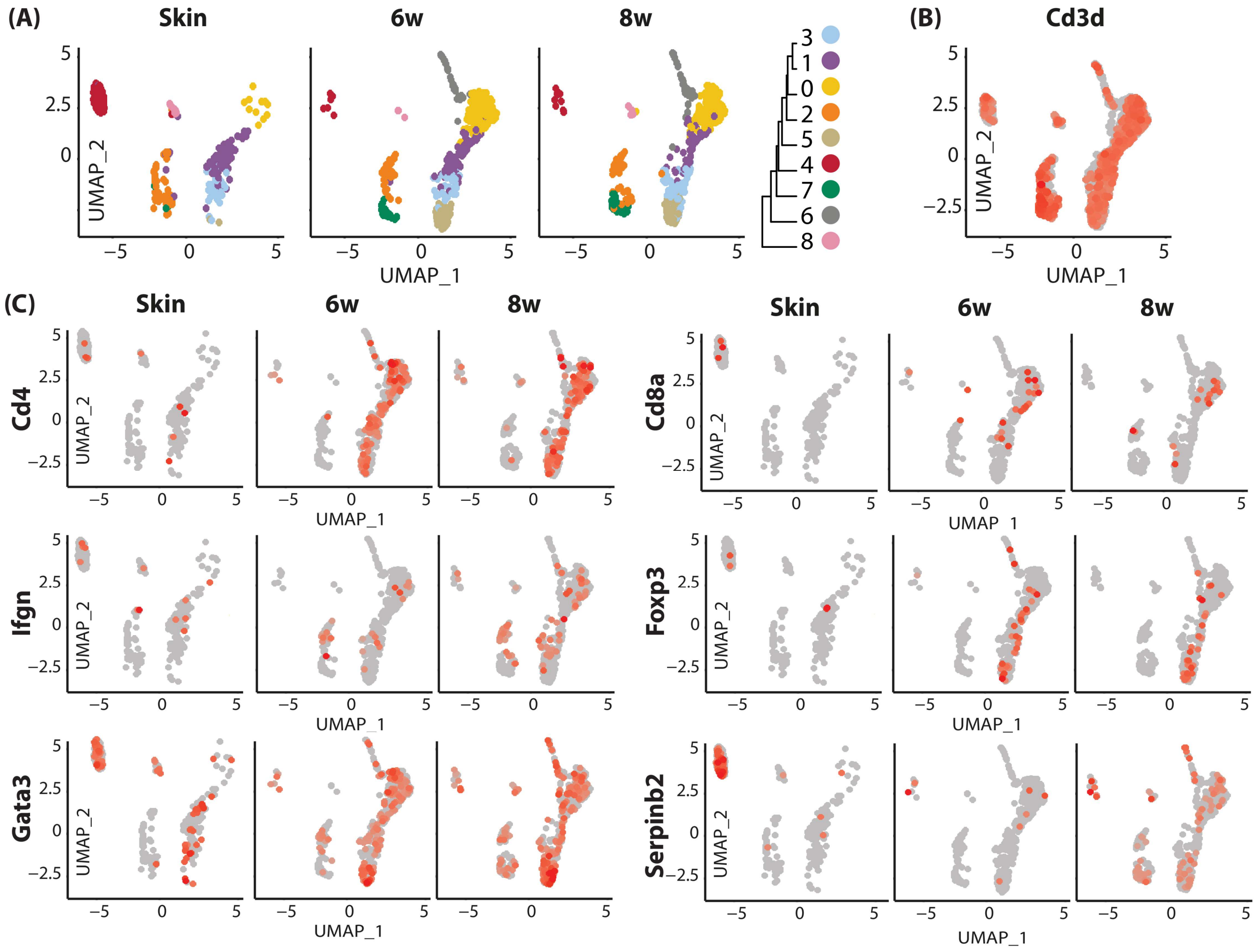

Figure S12: T-cell analysis in mouse scars

A) Subset of t-cells in mouse scar by unsupervised UMAP-clustering, split by timepoint. B) Feature plot of Cd3d in mouse t-cell subset. C) Feature plots of *Cd4* (cluster of differentiation 4), *Cd8a* (cluster of differentiation 4), *Ifgn* (interferon gamma), *Foxp3* (forkhead box P3), *Gata3* (GATA Binding Protein 3) and *Serpinb2* (Serpin Family B Member 2), split by timepoint. In feature plots, normalized log expression of the respective gene is mapped onto the UMAP-plot.

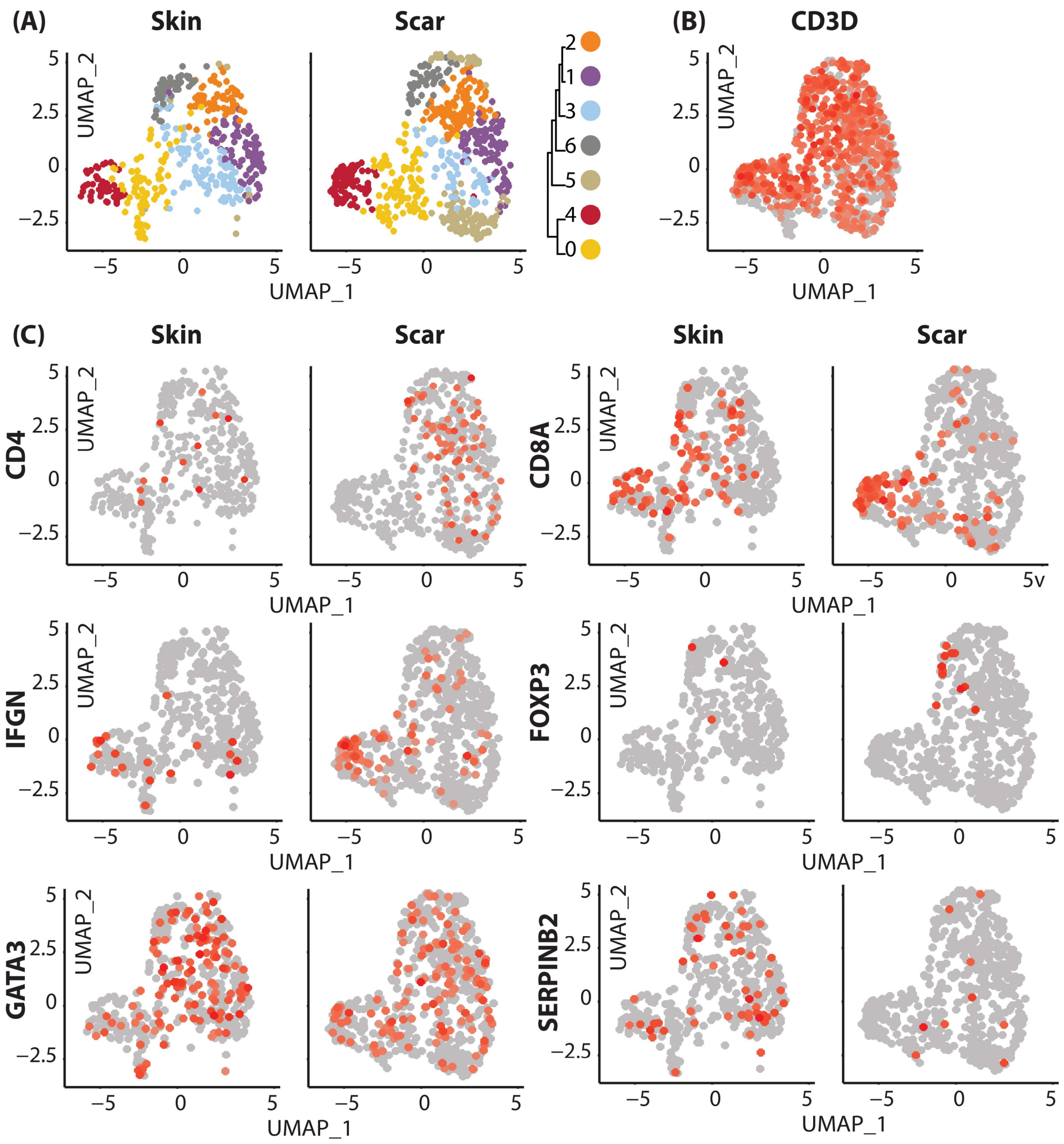

Figure S13: T-cell analysis in human scars.

A) Subset of t-cells in human scar by unsupervised UMAP-clustering, split by tissue. B) Feature plots of *CD3D* in human t-cell subset. C) Feature plots of *CD4* (cluster of differentiation 4), *CD8A* (cluster of differentiation 4), *IFGN* (interferon gamma), *FOXP3* (forkhead box P3), *GATA3* (GATA Binding Protein 3) and *SERPINB2* (Serpin Family B Member 2), split by tissue. In feature plots, normalized log expression of the respective gene is mapped onto the UMAP-plot.

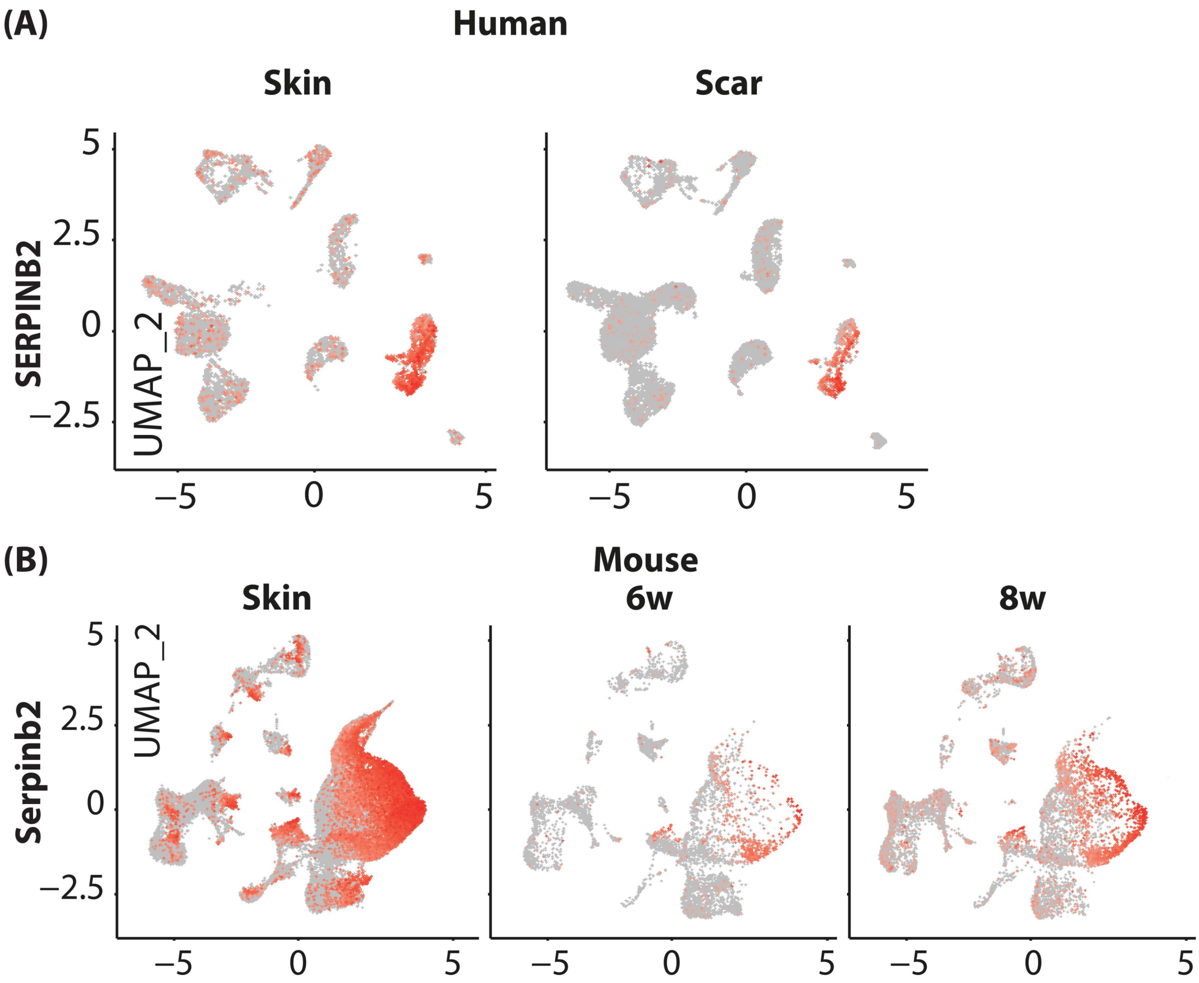

Figure S14: SERPINB2-expression in human and mouse.

Feature plots of *SERPINB2* (Serpin Family B Member 2) in A) human hypertrophic scar and skin and, split by tissue. B) mouse skin, 6 and 8 weeks old scars, split by timepoint. In feature plots, normalized log expression of the respective gene is mapped onto the UMAP-plot.
