# Supplementary material for: The serine proteases dipeptidyl-peptidase 4 and urokinase are key molecules in human and mouse scar formation
Source: Nat Commun. 2021 Oct 29;12:6242. doi: 10.1038/s41467-021-26495-2 (PMC8556235; doi:10.1038/s41467-021-26495-2)
Supplement: Supplementary file 3 — Reporting Summary [file 41467_2021_26495_MOESM3_ESM.pdf]

nature research

Corresponding author(s): Michael Mildner

Last updated by author(s): Aug 27, 2021

Reporting Summary

Nature Research wishes to improve the reproducibility of the work that we publish. This form provides structure for consistency and transparency in reporting. For further information on Nature Research policies, see our Editorial Policies and the Editorial Policy Checklist.

Statistics

For all statistical analyses, confirm that the following items are present in the figure legend, table legend, main text, or Methods section.

n/a	Confirmed	
☐	☒	The exact sample size (*n*) for each experimental group/condition, given as a discrete number and unit of measurement
☐	☒	A statement on whether measurements were taken from distinct samples or whether the same sample was measured repeatedly
☐	☒	The statistical test(s) used AND whether they are one- or two-sided<br>*Only common tests should be described solely by name; describe more complex techniques in the Methods section.*
☐	☒	A description of all covariates tested
☐	☒	A description of any assumptions or corrections, such as tests of normality and adjustment for multiple comparisons
☐	☒	A full description of the statistical parameters including central tendency (e.g. means) or other basic estimates (e.g. regression coefficient) AND variation (e.g. standard deviation) or associated estimates of uncertainty (e.g. confidence intervals)
☐	☒	For null hypothesis testing, the test statistic (e.g. *F*, *t*, *r*) with confidence intervals, effect sizes, degrees of freedom and *P* value noted<br>*Give P values as exact values whenever suitable.*
☒	☐	For Bayesian analysis, information on the choice of priors and Markov chain Monte Carlo settings
☒	☐	For hierarchical and complex designs, identification of the appropriate level for tests and full reporting of outcomes
☒	☐	Estimates of effect sizes (e.g. Cohen's *d*, Pearson's *r*), indicating how they were calculated

Our web collection on statistics for biologists contains articles on many of the points above.

Software and code

Policy information about availability of computer code

Data collection	For single-cell RNAseq (scRNAseq)-analysis, unwanted variations and low-quality cells were filtered by removing cells with high and low (>3000 and <200) Unique Molecular Identifier (UMI)-counts. First, healthy skin and scar samples were integrated separately to avoid clustering according to donors, and for batch correction. Subsequently, skin and scar data were integrated again into one dataset. Data integration was performed according to the recommended workflow by Butler et al. and Stuart et al. For identification of differentially expressed genes (DEGs), normalized count numbers were used, including genes present in the integrated dataset to avoid calculation of batch effects. As keratin and collagen genes were previously found to contaminate skin biopsy datasets and potentially provide a false-positive signal 34, these genes (COL1A1, COL1A2, COL3A1 and KRT1 KRT5, KRT10, KRT14, KRTDAP) were excluded from DEG calculation in non-fibroblast clusters (collagens) or non-keratinocyte clusters (keratins), respectively. Moreover, genes Gm42418, Gm17056 and Gm26917 caused technical background noise and batch effect in mouse scRNAseq, as described before 35, and were thus excluded from the dataset.<br><br>GO-tems were calculated in Cytoscape ClueGO with medium GO-specificity, with GO-term fusion, and only significant (P value <.05) were displayed.
Data analysis	Raw scRNAseq sequencing files were demultiplexed, aligned to the human or mouse reference genome (GrCh38/ mm10) and counted using the Cellranger pipelines (Cellranger v3, 10X Genomics). The resulting cell-gene matrices were processed using the 'Seurat'-package (Seurat v3.1.0, Satija Lab, New York, NY, USA) in R-studio in R (R v3.6.2, The R Foundation, Vienna, Austria).<br><br>Pseudotime analyses, trajectory-construction and calculation of pseudotime-dependent gene expression were performed in Monocle 2 (Monocle2, v2.14.0, Trapnell Lab, University of Washington, Seatlle, WA, USA)<br><br>As input for pseudotime ordering, differentially expressed genes between skin and scar were used, and trajectories were constructed with DDRTree (R-package 'DDRTree' v0.1.5, by Qi Mao, Li Wang et al., 2015)

ClueGO v2.5.5 41 plug-in in Cytoscape v3.7.2 was used for construction of GO-networks.

For statisical analyses, GraphPad Prism v8.0.1 (GraphPad Software, San Diego, USA) was used.
Fluorescence values of immunofluorescence stainings were analysed in ImageJ 1.53c in Java 1.8.0_172.
Blot volume analyses were performed with the Volume tool in ImageLab 6.0.1 (BioRad).
Primers were designed using the Primer3 software (version 0.4.0, https://bioinfo.ut.ee/primer3-0.4.0/).
Collagen alignment in H&E samples has been calculated according to Curvealign V4.0 Beta manual (August 31,2017).

For manuscripts utilizing custom algorithms or software that are central to the research but not yet described in published literature, software must be made available to editors and reviewers. We strongly encourage code deposition in a community repository (e.g. GitHub). See the Nature Research guidelines for submitting code & software for further information.

Data

Policy information about availability of data

All manuscripts must include a data availability statement. This statement should provide the following information, where applicable:

- Accession codes, unique identifiers, or web links for publicly available datasets
- A list of figures that have associated raw data
- A description of any restrictions on data availability

Figures 1-6 involve raw data of single-cell transcriptome sequencing (scRNAseq).
The scRNAseq data generated in this study have been deposited in the NCBI GEO database under accession GSE156326 [https://www.ncbi.nlm.nih.gov/geo/query/acc.cgi?acc=GSE156326, DOI]. The raw sequencing data are protected and are not publicly available due to data privacy laws. If raw data are absolutely necessary for further analyses or similar, they will be made available to other researchers upon reasonable request to the first author of this work (vera.vorstandlechner@meduniwien.ac.at), and will be provided within a two weeks time frame. All other source data are provided as a Source Data file.

For demultiplexing and genome alignment for scRNAseq-data, reference genomes GrCh38 (human) and mm10 (mouse) were used in this work.

Field-specific reporting

Please select the one below that is the best fit for your research. If you are not sure, read the appropriate sections before making your selection.

- [x] Life sciences
- [ ] Behavioural & social sciences
- [ ] Ecological, evolutionary & environmental sciences

For a reference copy of the document with all sections, see nature.com/documents/nr-reporting-summary-flat.pdf

Life sciences study design

All studies must disclose on these points even when the disclosure is negative.

Sample size	As the present study is a pilot study assessing hypertrophic scar tissue on scRNAseq-level, a sample size calculation based on expected effect size was not possible. A sample size of n=3 of skin and scar was chosen to allow comparability and screen for outliers in clustering and gene regulation in scRNAseq. For in vitro and mouse in vivo experiments, estimated effect sizes were not known, thus sample size calculation not possible. All sample sizes of the protein inhibitor mouse experiment, human primary cells, and human tissue donors were chosen with at least 4-5 per group to allow calculation of median, standard deviation and range.
Data exclusions	In scRNAseq datasets, cells that did not meet quality control standards (as described above) were excluded from analysis. Otherwise, no data was excluded.
Replication	To enable replication of experiments in in vitro primary human cells, standardized conditions were applied, and are stated in the methods section as detailed as possible. Primary skin fibroblast stimulation experiments were performed on five donors and was replicated successfully thrice.<br><br>scRNAseq-experiments were performed strictly according to standardized protocol. However, exact replication on scRNAseq is not possible, as the exact same sample (scar tissue) cannot be analysed repeatedly from the same patient.<br>Bioinformatic tools as described above allow calculation of batch of donor effects in scRNAseq, however, results of comparing skin with scar may still vary, based on age, ethnicity, and scar etiology of the donor population.<br><br>Mouse experiments were performed once as permitted by the ethics committee. They were conducted strictly according to protocol to enable exact replication by other researchers.
Randomization	Randomization of human skin/scar samples was not performed, samples were analyzed as available from the respective surgeries.<br><br>Mice were allocated randomly to study groups, with avoidance of grouping to littermates or previous housing.
Blinding	No blinding was performed in this study. Covariates (e.g. in application of protein inhibitors) were controlled by double-check-principle in all experimental steps, and data analysis of staining images, blots, ELISAs etc. was each performed by two independent researchers.

Reporting for specific materials, systems and methods

We require information from authors about some types of materials, experimental systems and methods used in many studies. Here, indicate whether each material, system or method listed is relevant to your study. If you are not sure if a list item applies to your research, read the appropriate section before selecting a response.

Materials & experimental systems

n/a	Involved in the study
☐	☒ Antibodies
☒	☐ Eukaryotic cell lines
☒	☐ Palaeontology and archaeology
☐	☒ Animals and other organisms
☐	☒ Human research participants
☒	☐ Clinical data
☒	☐ Dual use research of concern

Methods

n/a	Involved in the study
☒	☐ ChIP-seq
☒	☐ Flow cytometry
☒	☐ MRI-based neuroimaging

Antibodies

Antibodies used

Target -Supplier-Product Nr.-Host species-Dilution- Application (WB= Western blotting, IF=immunoflourescence, FFPE=formalin-fixed paraffin-embedded)

DPP4 - abcam - ab215711 - rabbit monoclonal -1:1000 IF (FFPE) -WB
PLAU - Novus biologicals - NBP2-20819 - rabbit polyclonal 1:100 IF (FFPE), WB
αSMA - abcam - ab7817 - mouse monclonal - 1:200 WB
Col3a1 - abcam - ab7778 - rabbit polyclonal 1:200 IF (FFPE)
Fibronectin-abcam - ab2413 - rabbit polyclonal 1:500 IF (FFPE)
GAPDH - abcam - ab8245 - mouse monclonal - 1:10000 - WB
pSMAD2 - Cell signaling - mAb 5339 - rabbit polyclonal - 1:1000 - WB
SMAD2/3 Cell signaling Rabbit mAb 8685 rabbit monoclonal 1:500 WB
pSMAD1/5/9 - Cell signaling - mAb 13820 - rabbit polyclonal - 1:1000 - WB
ERK 1/2 Cell signaling Rabbit mAb mAb4695 rabbit polyclonal 1:1000 WB
pERK1/2 - Cell signaling - mAb 4376 - rabbit polyclonal - 1:1000 - WB
Alexa fluor® - 546 anti-mouse IgG (H + L) - Invitrogen A-11030 goat polyclonal 1:500 IF, 2nd step
Alexa fluor® 546 - anti-rabbit IgG (H + L) -Invitrogen -A-11035 goat polyclonal - 1:500 IF, 2nd step
Anti-mouse, HRP-conjugated - GE Healthcare - GENX-A931 goat polyclonal - 1:10 000 - WB, 2nd step
Anti-rabbit, HRP-conjugated Bio-Rad - #1706515 - goat polyclonal - 1:10 000 - WB, 2nd step

Validation

All antibodies were used with respective 2nd step species IgG isotype antibodies as negative control and appropiate tissue samples as recommended by the manufacturers as positive control. All antibodies were only used for the respective species as recommended and validated by the manufacturer.

DPP4 ab215711:
Lindgren O et al. Gliptin-associated Bullous Pemphigoid and the Expression of Dipeptidyl Peptidase-4/CD26 in Bullous Pemphigoid. Acta Derm Venereol 99:602-609 (2019).
Zhao X et al. Long noncoding RNA CA7-4 promotes autophagy and apoptosis via sponging MIR877-3P and MIR5680 in high glucose-induced vascular endothelial cells. Autophagy 16:70-85 (2020).

PLAU - NBP2-20819
Manufacturer: Recombinant protein encompassing a sequence within the center region of human Urokinase. The exact sequence is proprietary.Target Molecular weight: 49kDa, WB-validation: u-Plasminogen Activator/Urokinase Antibody [NBP2-20819] - Sample (30 ug of whole cell lysate) A: IMR32 10% SDS PAGE diluted at 1:2000

Col3a1 ab7778:
ab7778 has been referenced in 416 publications.
Bochon K et al. The Effect of L-Ascorbic Acid and Serum Reduction on Tenogenic Differentiation of Human Mesenchymal Stromal Cells. Int J Stem Cells 14:33-46 (2021).
He J et al. Mechanical stretch promotes hypertrophic scar formation through mechanically activated cation channel Piezo1. Cell Death Dis 12:226 (2021).

alphaSMA ab7817:
ab7817 has been referenced in 498 references.
Cai X et al. miR-124a enhances therapeutic effects of bone marrow stromal cells transplant on diabetic nephropathy-related epithelial-to-mesenchymal transition and fibrosis. J Cell Biochem 121:299-312 (2020).

Fibronectin-abcam ab2413:
ab2413 has been referenced in 597 publications.
Hahn HM et al. The Effects of Subcutaneously Injected Novel Biphasic Cross-Linked Hyaluronic Acid Filler: In Vivo Study. Aesthetic Plast Surg 45:322-331 (2021).

GAPDH ab8245:
ab8245 has been referenced in 3285 publications.
Lin Q et al. Circular RNA ITCH downregulates GLUT1 and suppresses glucose uptake in melanoma to inhibit cancer cell proliferation. J Dermatolog Treat 32:231-235 (2021).
Meng QH et al. Circ_0000388 Exerts Oncogenic Function in Cervical Cancer Cells by Regulating miR-337-3p/TCF12 Axis. Cancer Biother Radiopharm 36:58-69 (2021).
Lu S et al. Trimetazidine alleviates hypoxia/reoxygenation-induced apoptosis in neonatal mice cardiomyocytes via up-regulating HMGB1 expression to promote autophagy. J Recept Signal Transduct Res 41:170-179 (2021).

pSMAD2 mAb 5339
mAb 5339 has been referenced in 264 publications.
Sun WY, Gu YJ, Li XR, et al. β-arrestin2 deficiency protects against hepatic fibrosis in mice and prevents synthesis of extracellular matrix. Cell Death Dis. 2020;11(5):389. Published 2020 May 21. doi:10.1038/s41419-020-2596-8

SMAD 2/3 mAb 8685
mAb 8685 has been referenced by 283 publications.
Proc Natl Acad Sci USA, 2021 Jun 22;118(25):e2023537118.
doi: 10.1073/pnas.2023537118.Addiction to Golgi-resident PI4P synthesis in chromosome 1q21.3-amplified lung adenocarcinoma cells

SMAD 1/5/9
Phospho-Smad1 (Ser463/465)/ Smad5 (Ser463/465)/ Smad9 (Ser465/467) (D5B10) Rabbit mAb recognizes endogenous levels of Smad1 and Smad5 protein when phosphorylated at Ser463/465 and Smad9 (Smad8) protein when phosphorylated at Ser465/467.
mAb 13820 has been referenced by 190 publications
J Transl Med. 2021 Jan 20;19(1):37. doi: 10.1186/s12967-020-02654-9. Reduction of miR-744 delivered by NSCLC cell-derived extracellular vesicles upregulates SUV39H1 to promote NSCLC progression via activation of the Smad9/BMP9 axis

ERK1/2 mAb 4695
p44/42 MAP Kinase (137F5) Rabbit mAb detects endogenous levels of total p44/42 MAP kinase (Erk1/Erk2) protein. The antibody does not cross-react with JNK/SAPK or p38 MAP kinase.
mAb 4695 has been referenced by 3679 publications.
Bioengineered. 2021 Dec;12(1):30-43. doi: 10.1080/21655979.2020.1855914. Guifu Wang :GIT1 overexpression promotes epithelial-mesenchymal transition and predicts poor prognosis in hepatocellular carcinoma

pERK1/2 mAb 4376
mAb 4376 has been referenced by 717 publications.
Baumann D, Hägele T, Mochayedi J, et al. Proimmunogenic impact of MEK inhibition synergizes with agonist anti-CD40 immunostimulatory antibodies in tumor therapy. Nat Commun. 2020;11(1):2176. Published 2020 May 1. doi:10.1038/s41467-020-15979-2

Alexa fluor® - 546 anti-mouse IgG A-11030
A-11030 has been referenced by 161 publications in western blotting, immunohistochemistry paraffin/frozen and miscellaneous.
Nat Commun. 2019 Jul 24;10(1):3304. doi: 10.1038/s41467-019-11093-0. ERAP1 promotes Hedgehog-dependent tumorigenesis by controlling USP47-mediated degradation of βTrCP

Alexa fluor® 546 - anti-rabbit IgG A-11035
A-11035 has been referenced by 158 publications in western blotting, immunohistochemistry paraffin/frozen and miscellaneous.
Nat Cell Biol. 2018 Oct;20(10):1193-1202.doi: 10.1038/s41556-018-0179-z. Epub 2018 Sep 3.Stem cell functionality is microenvironmentally defined during tumour expansion and therapy response in colon cancer

Anti-mouse, HRP-conjugated GGENX-A931
Ali E Sifat: Nicotine and electronic cigarette (E-Cig) exposure decreases brain glucose utilization in ischemic stroke.
Journal of neurochemistry (2018-08-01), PMID30062776

Anti-rabbit, HRP-conjugated Bio-Rad - #1706515
Manufacturer: Double-affinity purified, Human IgG adsorbed

Animals and other organisms

Policy information about studies involving animals; ARRIVE guidelines recommended for reporting animal research

Laboratory animals	female Balb/c mice, 6-8 weeks old
Wild animals	No wild animals were used in this study.
Field-collected samples	This study did not involve field-collected samples.
Ethics oversight	Animal experiments were approved by the Medical University of Vienna ethics committee and by the Austrian Federal Ministry of Education, Science and Research (Vote Nr. BMBWF-66.009/0075-V/3b/2018) and performed in accordance with the Austrian guidelines for the use and care of laboratory animals.

Note that full information on the approval of the study protocol must also be provided in the manuscript.

Human research participants

Policy information about studies involving human research participants

Population characteristics	Resected scar tissue was obtained from patients who underwent elective scar resection surgery (donor information is provided in Table S1). Scars were classified as hypertrophic, pathological scars according to POSAS 30 by a plastic surgeon. Only mature scars, which had not been treated before and persisted for more than two years were used for all experiments. All donors had no known chronic diseases and received no chronic medication. Healthy skin was obtained from female donors between 25-45 years from surplus abdominal skin removed during elective abdominoplasty.
Recruitment	Participants were recruited upon admission for surgery to the Division of Plastic Surgery, Department of Surgery, Medical University of Vienna. Patients undergoing suitable surgery for removal of skin or scars were recruited and provided written informed consent. Recruitment bias may be present, as only patients with medically significant scar (i.e. painful, impacting mobility and life quality) are admitted for surgical treatment. Samples gained from other hypertrophic scars that are not significantly impairing patient's and life quality, and thus not admitted for surgical treatment, might yield different results.
Ethics oversight	The Vienna Medical University ethics committee approved the use of healthy abdominal skin (Vote Nr. 217/2010) and of scar tissue (Vote Nr. 1533/2017) and all donors provided written informed consent.

Note that full information on the approval of the study protocol must also be provided in the manuscript.
